# Insights from Hi-C data regarding the Pacific salmon louse (*Lepeophtheirus salmonis*) sex chromosomes

**DOI:** 10.1093/g3journal/jkae087

**Published:** 2024-04-29

**Authors:** Anne-Marie Flores, Kris A Christensen, Ahmed Siah, Ben F Koop

**Affiliations:** Department of Biology, University of Victoria, Victoria, BC V8W 2Y2, Canada; Department of Biology, University of Victoria, Victoria, BC V8W 2Y2, Canada; British Columbia Centre for Aquatic Health Sciences, Campbell River, BC V9W 2C2, Canada; Department of Biology, University of Victoria, Victoria, BC V8W 2Y2, Canada

**Keywords:** sea lice, Pacific, farmed salmon, ocean, parasite, ZW sex determination

## Abstract

Salmon lice, *Lepeophtheirus salmonis* (family Caligidae), are ectoparasites that have negatively impacted the salmon aquaculture industry and vulnerable wild salmon populations. Researchers have studied salmon lice to better understand their biology to develop effective control strategies. In this study, we updated the chromosome-level reference genome assembly of the Pacific subspecies of *L. salmonis* using Hi-C data. The previous version placed contigs/scaffolds using an Atlantic salmon louse genetic map. By utilizing Hi-C data from Pacific salmon lice, we were able to properly assign locations to contigs/scaffolds previously unplaced or misplaced. This resulted in a more accurate genome assembly and a more comprehensive characterization of the sex chromosome unique to females (W). We found evidence that the same ZW-ZZ mechanism is common in both Atlantic and Pacific subspecies of salmon lice using PCR assays. The W chromosome was approximately 800 kb in size, which is ∼30 times smaller than the Z chromosome (24 Mb). The W chromosome contained 61 annotated genes, including 32 protein-coding genes, 27 long noncoding RNA (lncRNA) genes, and 2 pseudogenes. Among these 61 genes, 39 genes shared homology to genes found on other chromosomes, while 20 were unique to the W chromosome. Two genes of interest on the W chromosome, prohibitin-2 and kinase suppressor of ras-2, were previously identified as potential sex-linked markers in the salmon louse. However, we prioritized the 20 unique genes on the W chromosome as sex-determining candidates. This information furthers our understanding of the biology of this ectoparasite and may help in the development of more effective management strategies.

## Introduction

The salmon louse (*Lepeophtheirus salmonis*) is a copepod species in the Caligidae family, commonly known as sea lice. This ectoparasite feeds on the mucus, skin, blood, and underlying tissue of salmonid species, notably Pacific salmon and trout, in marine environments (Johnson *et al.* 2004; Boxaspen 2006). This feeding behavior can lead to skin damage, reduced growth, osmotic imbalance, immunosuppression, and bacterial coinfections in salmonid hosts, which consequently increases the risk of mortality (Pike and Wadsworth 1999; Tully and Nolan 2002; Fjelldal *et al.* 2019; Fjelldal *et al*. 2020).

Salmon lice infestations have been associated with numerous disease outbreaks and significant economic losses in the salmon aquaculture industry and have been correlated with the decline in vulnerable wild salmonid populations (Pike and Wadsworth 1999; Costello 2006, 2009). Notably, multiple species of sea lice have been observed on numerous Pacific salmon species within the coastal waters of British Columbia, Canada (Beamish *et al.* 2005).

To mitigate the potentially adverse effects of salmon lice on both salmon restoration efforts and the economy, genetic research is ongoing (Boxaspen 2006; Yasuike *et al.* 2012). This research has resulted in the production of high-density genetic maps, transcriptomes, and whole-genome resequencing data (Yazawa *et al.* 2008; Besnier *et al.* 2014; Messmer *et al.* 2018; Danzmann *et al.* 2019), aimed at enhancing our understanding of salmon lice biology and ecology and at developing more effective control strategies. Genome assemblies were also generated for both the Pacific and Atlantic allopatric subspecies of the salmon louse (Messmer *et al*. 2018; Skern-Mauritzen *et al*. 2021; Joshi *et al*. 2022).

Estimates of the salmon louse genome size varied from 665 to 790 Mb (632–790 Mb as of writing) through sequencing techniques and 597–1,600 Mb with cytometric techniques (Wyngaard *et al.* 2022). From these and related studies, researchers identified 14 autosomes in the salmon louse, and that sex determination followed a genetic ZW-ZZ system (Carmichael *et al.* 2013; Besnier *et al.* 2014; Skern-Mauritzen *et al.* 2021). Furthermore, a sex-linked genetic marker within the coding region of the prohibitin-2 gene was isolated, although its functional role in sex determination was unclear (Carmichael *et al*. 2013; Messmer *et al*. 2018; Borchel *et al*. 2022).

The most recent reference genome assembly (contig N50: ∼4.5 Mb) was generated by placing Pacific salmon louse contigs onto pseudochromosomes utilizing an Atlantic salmon louse genetic map (Danzmann *et al*. 2019; Joshi *et al.* 2022). In this study, we generated Hi-C data from a Pacific salmon louse to use for placing contigs instead. This improves the genome assembly by reducing order and orientation issues caused by using a genetic map from a different subspecies (Skern-Mauritzen *et al.* 2014). We were also able to better characterize the female-specific W chromosome.

## Materials and methods

### Sampling

As in Joshi *et al.* (2022), Pacific Ocean salmon lice were collected from an Atlantic salmon farm in March 2022 by employees of the British Columbia Centre for Aquatic Health Sciences. The salmon farm is located near Vancouver Island in British Columbia, Canada. Samples were flash frozen on dry ice until they could be stored at −80°C. Female salmon lice were used for the Hi-C library preparation.

### Hi-C library

A Hi-C library was generated by Canada's Michael Smith Genome Sciences Centre (Vancouver, BC, Canada) using the Arima-HiC 2.0 kit (Arima Genomics) according to the manufacturers' instructions. Library products were amplified with 10 reaction cycles using NEBNext Q5 Master Mix (New England Biolabs) supplemented with 2 mM MgSO_4_. The library was sequenced on the Illumina NovaSeq platform using PE150 sequencing (NCBI accession: SRR24288523).

### Genome assembly

We mapped Hi-C reads to the genome assembly produced and submitted to the National Center for Biotechnology Information (NCBI) by Joshi *et al*. (2022). Contamination previously identified by the NCBI was removed from the genome assembly. Mapping the Hi-C reads to the genome assembly was performed using scripts from Arima Genomics (“mapping_pipeline” 2023). We converted the alignments to Hi-C format using the Matlock program (https://github.com/phasegenomics/matlock) and sorted the links produced by this program using Unix commands. The initial order and orientation of scaffolds was taken from the Joshi *et al*. (2022) AGP file, converted to assembly format (https://github.com/phasegenomics/juicebox_scripts), and the links were remapped using 3D DNA (Dudchenko *et al.* 2017). The previous order and orientation was then reviewed in Version 1.11.08 of Juicebox (Rao *et al.* 2014; Durand *et al.* 2016). In Juicebox, we manually ordered and oriented the scaffolds. The final assembly was output using scripts from Phase Genomics (https://github.com/phasegenomics/juicebox_scripts).

### W chromosome and sex determination

The NCBI annotated the genome assembly from Joshi *et al.* (2022), and these annotations were utilized to identify genes on the W chromosome. We used BLAST to identify if there were homologous genes on other chromosomes (Altschul *et al.* 1990). Potentially, a sex-determining gene could be unique to the W chromosome, as SRY in the XX-XY system in mammals (Gubbay *et al.* 1990; Sinclair *et al.* 1990), or the sex-determining gene could be duplicates of an autosomal gene (Song *et al.* 2021). Since the W chromosome is smaller in size compared to the Z chromosome, analogous to the Y chromosome in mammals, we prioritized identifying genes that were unique to the W chromosome as sex-determining candidates. Homologous proteins and RNA sequences were interrogated with BLASTN (minimum query coverage: 85%, minimum percent identity: 50%) using default parameters. Genes with and without homologs were visualized using Circos (Krzywinski *et al.* 2009). Genes that were unique to the W chromosome were considered potential sex-determining genes.

### Unique W chromosome gene verification

To determine if these unique genes on the W chromosome could be used as sex-specific markers, we designed primers for a subset of the protein-coding genes (Supplementary Table 1) using NCBI Primer-BLAST (Ye *et al.* 2012). We tested the primers using isolated genomic DNA from phenotypically sexed adult males and females of the Pacific and Atlantic subspecies (Supplementary Table 2). Pacific salmon louse specimens were collected from 2 aquaculture sites on Vancouver Island, British Columbia, Canada, from 2010 to 2014, and the Atlantic salmon lice were collected off the western coast of Greenland in 2011. Pacific salmon lice samples were previously described in Messmer *et al.* (2018). In addition to these primers, we developed primers for the previously identified sex-linked marker Prohibitin-2 (Carmichael *et al*. 2013).

The cephalothorax was removed from salmon louse samples preserved in ethanol. The tissue was homogenized using the Qiagen TissueLyser, and DNA was extracted following the Qiagen supplementary protocol *Purification of total DNA from insects* using the DNeasy Blood and Tissue kit. DNA was amplified for each primer set using ProMega GoTaq Hot Start Polymerase using the reagents and thermocycling conditions described in Supplementary Table 3. A nontemplate control was included for each reaction. PCR products were visualized on 2% TAE agarose gels stained with SYBR safe. If the gene was sex specific, an amplified product of the correct size would be present in the females and absent in the male specimens. In total, 40 DNA samples were tested per primer set.

## Results and discussion

We produced an improved chromosome-level reference genome assembly of the Pacific subspecies of *L. salmonis* by placing Pacific salmon louse contigs using Hi-C data generated from the same subspecies. The new salmon louse genome assembly (UVic_Lsal_1.3) is 647 Mb long, comprised of 8,687 contigs (N50 = 4,499,711) and 8,329 scaffolds (N50 = 47,531,287). These metrics were similar to the genome assembly reported by Joshi *et al*. (2022) (Table 1).

**Table 1.** Genome sequencing and assembly results for the updated *L. salmonis* genome assembly (UVic Lsal_1.3) compared to the previous version (UVic_Lsal_1.2) generated by Joshi *et al*. (2022).

Assembly statistics	Current UVic_Lsal_1.3	Recently published UVic_Lsal_1.2
Total sequence length	647,212,781	647,191,672
Total ungapped length	647,177,035	647,131,226
Gaps between scaffolds	0	0
Number of scaffolds	8,329	8,066
Scaffold N50	47,531,287	48,457,437
Scaffold L50	6	6
Number of contigs	8,687	8,671
Contig N50	4,499,711	4,499,712
Contig L50	44	44
Total number of chromosomes	16	15
Number of component sequences	8,685	8,699

**Fig. 1.**
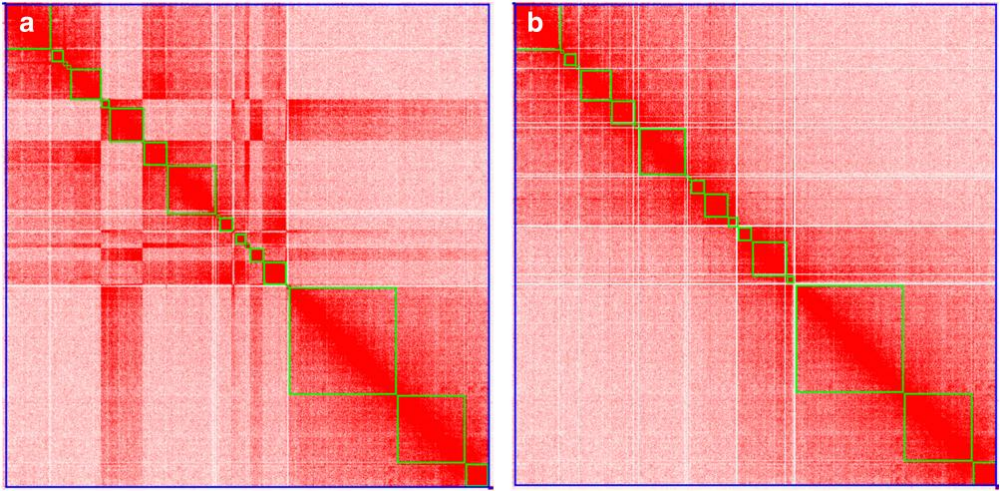
Juicebox contact map of chromosome 4 of the Pacific salmon louse. Contact map a) was produced using the previous genome assembly, which was scaffolded using an Atlantic salmon louse subspecies genetic map. Contact map b) of new genome assembly using the Pacific salmon louse Hi-C data.

**Fig. 2.**
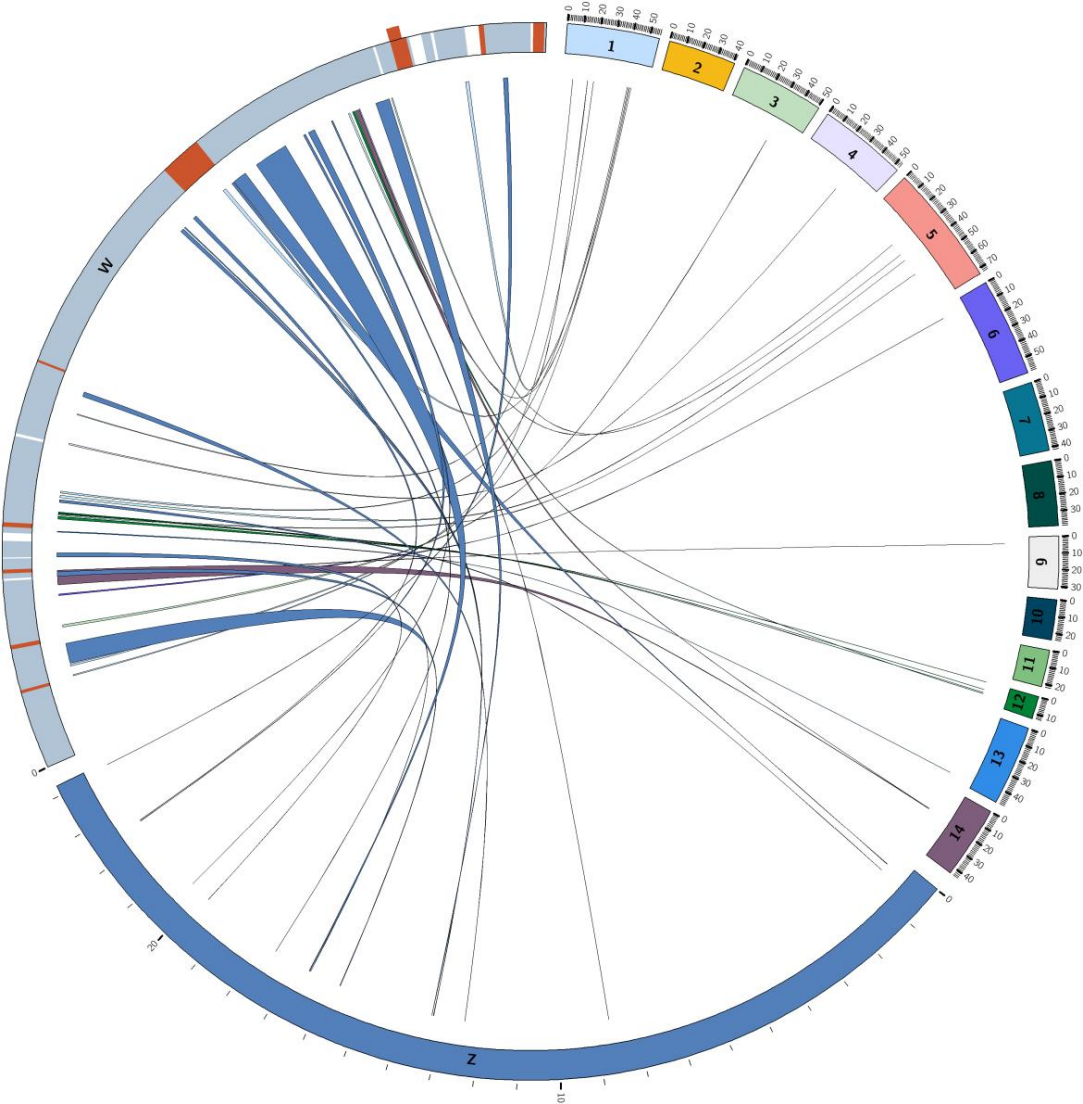
Circos plot of protein-coding and lncRNA genes on the W chromosome (800,023 bp) aligned to the remainder of the Pacific salmon louse genome assembly. Linkage groups with marks every million base pairs were drawn on the outer edge of the Circos plot. The W chromosome and Z chromosome were magnified by 800× and 26×, respectively, in order to display more detail. Protein-coding and lncRNA genes found on the W chromosome with a single ortholog or paralog in the remainder of the genome are shown with links between them. Potential sex-determining protein-coding genes (orange/darker shade) and lncRNA genes (white) are also indicated on the W chromosome and listed in order in Table 2. An overlapping protein-coding gene was placed on the outside of the main plot.

We were able to manually incorporate previously unplaced or misplaced contigs by using the Pacific salmon lice Hi-C data (Table 1 and Fig. 1). Chromosome-specific variations were observed; certain chromosomes exhibited minor changes, whereas others showed a significant improvement in the order and orientation of contigs (Fig. 1). Sixteen pseudochromosomes were constructed for the Pacific salmon louse, comprised of 14 autosomes and the Z and W sex chromosomes (Fig. 2).

In the previous genome assembly, 3 scaffolds were identified as part of the W chromosome (Joshi *et al*. 2022). This was based on the absence of these scaffolds in males and their high homology to genes found on the Z chromosome (Joshi *et al*. 2022). In this updated version, we successfully incorporated these scaffolds, along with several others, to the W chromosome (Table 2). These contigs were previously unplaced or incorrectly positioned on chromosome 13. Contigs that were misplaced on chromosome 13 are designated by an asterisk in Table 2.

**Table 2.** Contigs positioned on the W chromosome and the number of annotated genes present on each contig.

Accession	Number of genes present
JADKYV010006968	7
JADKYV010005591	18
JADKYV010004739*	5
JADKYV010006545	0
JADKYV010007195*	11
JADKYV010000915	19
JADKYV010006546	0
JADKYV010006547	0
JADKYV010006548	0
JADKYV010006549	0
JADKYV010007267	1

Gene annotations were based on the previous genome assembly (UVic_Lsal_1.2), as the most recent version (UVic_Lsal_1.3) was not annotated at the time of writing. Two contigs were misplaced on chromosome 13 in the previous version and are indicated by an asterisk (*).

The W chromosome was 800,023 bp in size, ∼30 times smaller than the Z chromosome (24,027,865 bp). The W chromosome contained 61 annotated genes, including 32 protein-coding genes, 27 long noncoding RNA (lncRNA) genes, and 2 pseudogenes (Supplementary Table 4). These annotations are based on version 1.2 of the salmon louse (UVic_Lsal_1.2) genome assembly, as the most recent genome (UVic_Lsal_1.3) remains unannotated at the time of writing. Among these 61 genes, 39 shared homology to genes found on other chromosomes, 2 were pseudogenes, while 20 genes were unique to the W chromosome (Fig. 2 and Table 3; Supplementary File 1).

**Table 3.** Potential sex-determining genes present on the W chromosome of the Pacific salmon louse.

Gene type	GeneID	Location	Gene description
Low-quality protein		NW_024052999.1 (58874..61041, complement)	luc7-like protein 3
**Protein**		**NW_024052999.1 (92704..95487, complement)**	**Uncharacterized protein LOC121131425**
lncRNA		NW_024051983.1 (33337..34615)	Uncharacterized LOC121131294
Protein		NW_024051983.1 (38865..41467)	Cilia- and flagella-associated protein 20
lncRNA		NW_024051983.1 (50013..50645)	Uncharacterized LOC121131298
lncRNA	Isoform X2	NW_024051983.1 (63372..69254, complement)	Uncharacterized LOC121131296
**Protein**	**Isoform X1**	**NW_024051983.1 (73937..77117, complement)**	**Uncharacterized protein LOC121131305**
lncRNA		NC_052143.1 (35813407..35815159)*	Uncharacterized LOC121128254
Protein		NC_052143.1 (35755653..35757282, complement)*	Transcription initiation factor TFIID subunit 8-like
Low-quality protein		NC_052143.1 (35922021..35953669)*	Mitogen-activated protein kinase kinase kinase 9-like
lncRNA		NW_024048138.1 (47782..49211, complement)	Uncharacterized LOC121130969
Protein		NW_024048138.1 (60968..70124)	Transcription factor Sox-7-like
**Protein**		**NW_024048138.1 (61226..73503)**	**Uncharacterized LOC121130980**
lncRNA		NW_024048138.1 (75749..84878, complement)	Uncharacterized LOC121130976
lncRNA		NW_024048138.1 (92303..94255)	Uncharacterized LOC121130982
lncRNA		NW_024048138.1 (118169..128671, complement)	Uncharacterized LOC121130985
Protein	Isoform X2	NW_024048138.1 (128797..132358)	Troponin I-like
lncRNA		NW_024048138.1 (168014..169600, complement)	Uncharacterized LOC121130977
Protein		NW_024048138.1 (169815..172782, complement)	G1/S-specific cyclin-E-like
**Protein**	**Isoform X1**	**NW_024048138.1 (173272..178007)**	**G1/S-specific cyclin-E-lik**

We identified homologous genes of W chromosome genes on other chromosomes using a blastn search on the NCBI. We considered a gene to be a sex-determining candidate if it only aligned to the W chromosome and no other location (criteria of homologous gene: query coverage ≥ 85%, percent identity ≥ 50%). Annotated genes were based on the previous genome assembly (UVic_Lsal_1.2)—2 contigs for the W chromosome were misplaced on chromosome 13 in the previous version and are indicated by an asterisk (*). The order of potential sex-determining genes is schematically shown on Fig. 2 (Circos plot). We designed primers for the protein-coding genes labeled in bold.

Two genes of interest located on the W chromosome, prohibitin-2 and kinase suppressor of ras-2, were previously identified as potential sex-specific markers in the salmon louse (Carmichael *et al*. 2013; Messmer *et al*. 2018). Our analysis revealed that both genes had homologs/orthologs on the Z chromosome, suggesting that these genes may not be sex determining. While it is possible to design sex-specific markers of the prohibitin-2 gene by targeting specific regions (Supplementary File 2; Carmichael *et al*. 2013; Messmer *et al*. 2018; Borchel *et al*. 2022), these genes are not specific to the W chromosome. We identified 20 genes unique to the W chromosome that are more promising sex-determining gene candidates.

The majority of the sex-specific genes were uncharacterized, but some were characterized. These included luc7-like protein 3, mitogen-activated protein kinase kinase kinase, G1/S-specific cyclin-E-like, cilia- and flagella-associated protein 20, transcription factor Sox-7-like, and troponin I-like. Several of these genes were previously reported as potential sex-specific markers and may have a functional role in sex determination (Messmer *et al*. 2018; Borchel *et al*. 2022).

We designed sex-specific markers for 4 of the sex-determining candidates, which included G1/S-specific cyclin E-like and 3 uncharacterized protein-coding genes. Using these genetic markers, we successfully identified the sex of our salmon lice samples for both subspecies across all markers (Supplementary File 2). Only 1 Atlantic male was not able to be sexed genetically using the G1/S-specific cyclin E-like marker and LOC121131305 primer set. This was caused by unsuccessful PCR amplifications. All 20 female samples amplified the W chromosome markers we designed, and 19 males did not across 3 Pacific and 1 Atlantic Ocean sampling locations. These findings suggest that these unique genes on the W chromosome can be used to determine the genetic sex of salmon lice, and this is a common mechanism between subspecies. Further research is necessary to determine the functional relationship of these genes and sex determination.

The identification of sex determination genetic pathways could be a component for the development of population suppression technologies (e.g. Siddall *et al*. 2022). Genetic pest management (GPM) is a technology that uses the natural mating system of the pest species to introduce into the target population traits that will either reduce their numbers or their ability to cause damage (Leftwich *et al.* 2021; Siddall *et al.* 2022). The most commonly used methods in GPM for suppression include the sterile insect technique (SIT; Knipling 1955), genetic modification (Siddall *et al*. 2022), or the use of gene drives (Champer *et al.* 2016). GPM shows promise for controlling pest populations in a more targeted and sustainable manner compared to traditional methods like chemical therapeutants while minimizing potential harm to the surrounding environment and nontarget species.

Developing alternative methods for controlling salmon lice is important since the aquaculture industry was over reliant on existing chemical treatments, resulting in resistance to the majority of available therapeutants (reviewed in Aaen *et al*. 2015). For example, resistance to emamectin benzoate (EMB) has been reported in all major Atlantic salmon farming industries around the world (Bravo *et al*. 2008; Lees *et al.* 2008; Jones *et al*. 2012; Lam *et al*. 2020; Godwin *et al*. 2022). Additionally, Poley *et al*. (2015) observed different reactions between male and female salmon lice to EMB; males demonstrated a higher tolerance to EMB compared to females (Poley *et al*. 2015). This emphasizes the pressing need to explore alternative, sustainable approaches for managing sea lice.

We updated the reference genome assembly for the Pacific salmon louse by reducing order and orientation issues caused by using a genetic map generated from Atlantic salmon lice. Our investigation led to a more comprehensive characterization of the W chromosome and the identification of 20 unique genes that are sex-determining candidates. However, further studies are essential to fully elucidate the functionalities of these genes in the context of sex determination.

## Supplementary Material

jkae087_Supplementary_Data

## Data Availability

The genome assembly is available in the NCBI database under the following accession number: GCA_016086655.4. The Hi-C raw reads are available under SRR24288523. Supplemental material available at G3 online.
