# Supplementary material for: Insights from Hi-C data regarding the Pacific salmon louse (Lepeophtheirus salmonis) sex chromosomes
Source: G3 (Bethesda). 2024 Apr 29;14(7):jkae087. doi: 10.1093/g3journal/jkae087 (PMC11228835; doi:10.1093/g3journal/jkae087)
Supplement: jkae087_Supplementary_Data [file jkae087_supplementary_data.zip › Supplemental_Material_Legends_G3-2024-404899.docx]

Table S1. Primers designed using NCBI Primer-BLAST for four protein-coding genes (G1/S-specific cyclin E-like and three uncharacterized protein coding genes) that were unique on the W-chromosome to determine if they could be used as sex-specific markers. Primers were also designed for the previously identified sex-linked marker Prohibitin-2.

Table S2. List of salmon louse specimens used to test our potential sex-determining markers. Genomic DNA was isolated from phenotypically sexed adult males and females of the Pacific and Atlantic subspecies. Pacific salmon louse specimens were collected from two aquaculture sites on Vancouver Island, British Columbia, Canada from 2010 – 2014, and the Atlantic salmon lice were collected off the western coast of Greenland in 2011. Pacific salmon lice samples were previously described in Messmer *et al*. (2018). In total, 40 samples were used per primer set.

Table S3. Reagents and thermocycling conditions used to amplify salmon louse gDNA for each primer set.

Table S4. List of genes identified on the Pacific salmon louse W-chromosome. The W-chromosome contained 61 annotated genes, including 32 protein-coding genes, 27 long non-coding RNA (lncRNA) genes, and 2 pseudogenes. These annotations are based on version 1.2 of the salmon louse (UVic_Lsal_1.2) genome assembly, as the most recent genome (UVic_Lsal_1.3) remains unannotated at the time of writing.

File S1. List of genes identified on the W-chromosome that share homology to genes found on other chromosomes or are unique to the W-chromosome. These annotations are based on version 1.2 of the salmon louse (UVic_Lsal_1.2) genome assembly, as the most recent genome (UVic_Lsal_1.3) remains unannotated at the time of writing.

File S2. PCR amplification results from four sex-determining candidates designed using NCBI Primer-BLAST. PCR products were visualized on a 2% agarose TAE agarose gels stained with SYBR safe. If the gene was sex-specific, an amplified product of the correct size would be present in the females, and absent in the male specimens. A non-template control (25S rRNA) was also used per reaction for both subspecies to determine if the PCR amplification was successful or failed. The PCR product length is described in Table S1. In total, 40 DNA samples were tested per primer set.
