# Supplementary figures and images for: Insights from Hi-C data regarding the Pacific salmon louse (Lepeophtheirus salmonis) sex chromosomes
Source: G3 (Bethesda). 2024 Apr 29;14(7):jkae087. doi: 10.1093/g3journal/jkae087 (PMC11228835; doi:10.1093/g3journal/jkae087)

## Slide 1
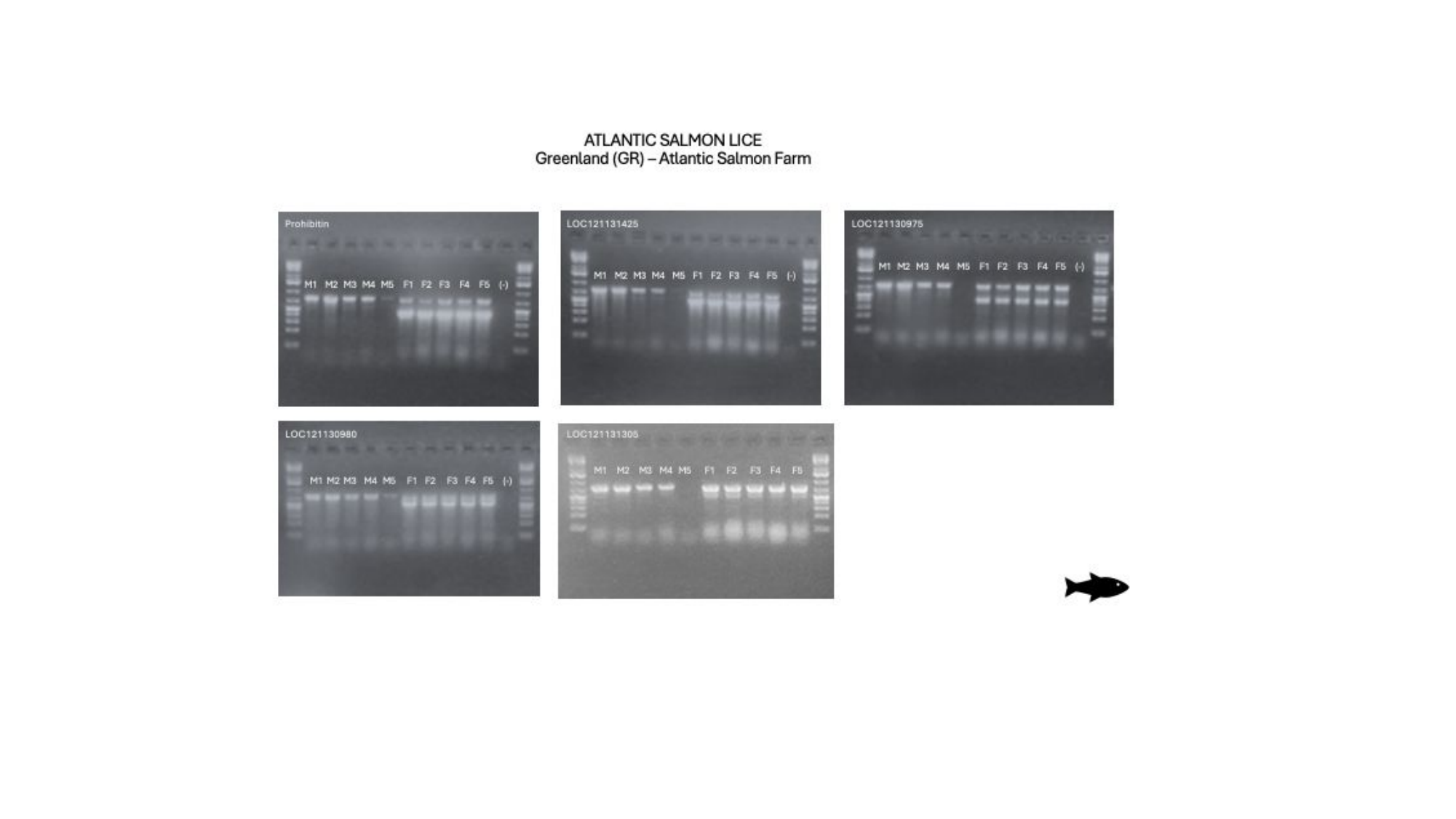

## Slide 2
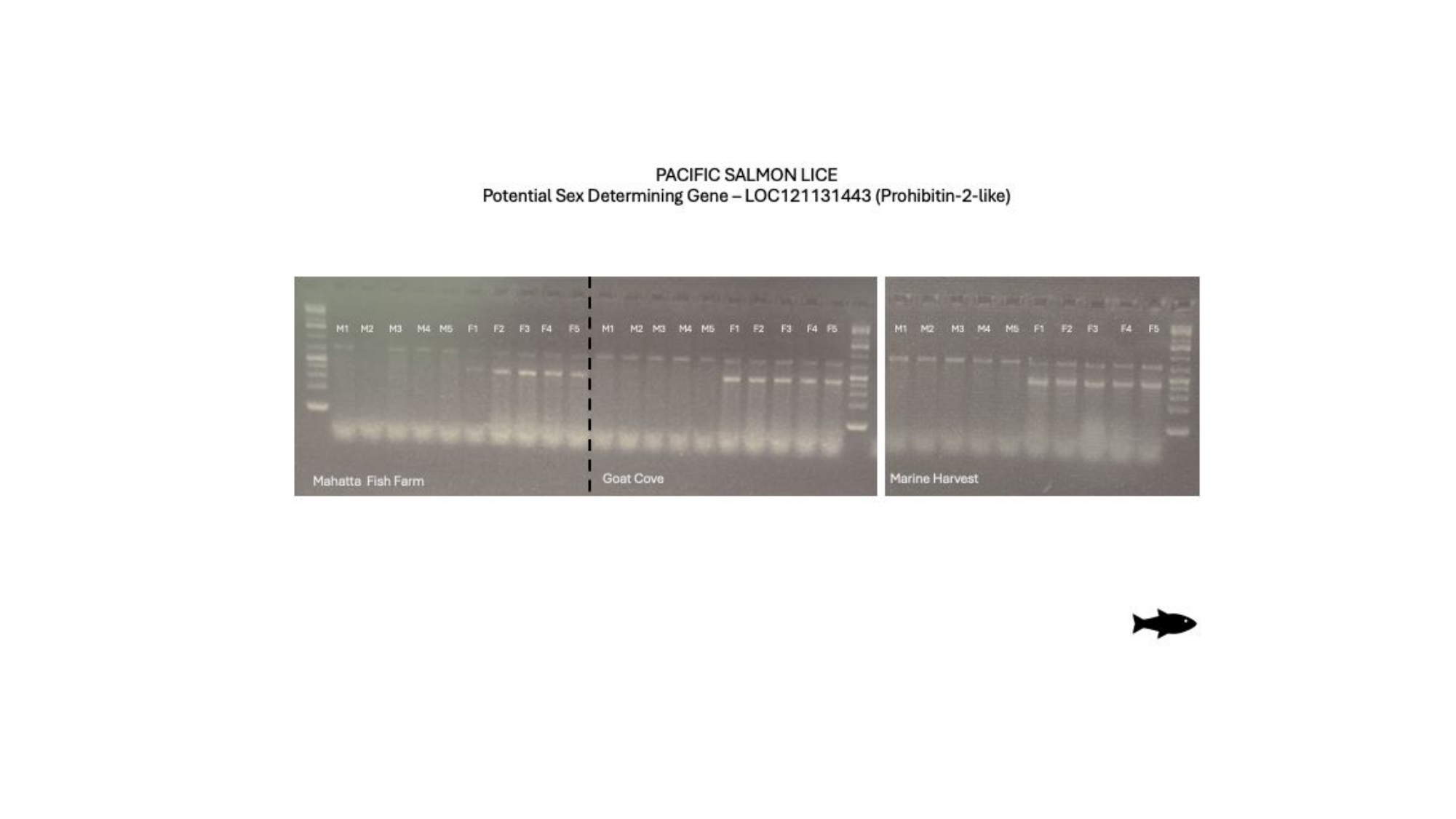

## Slide 3
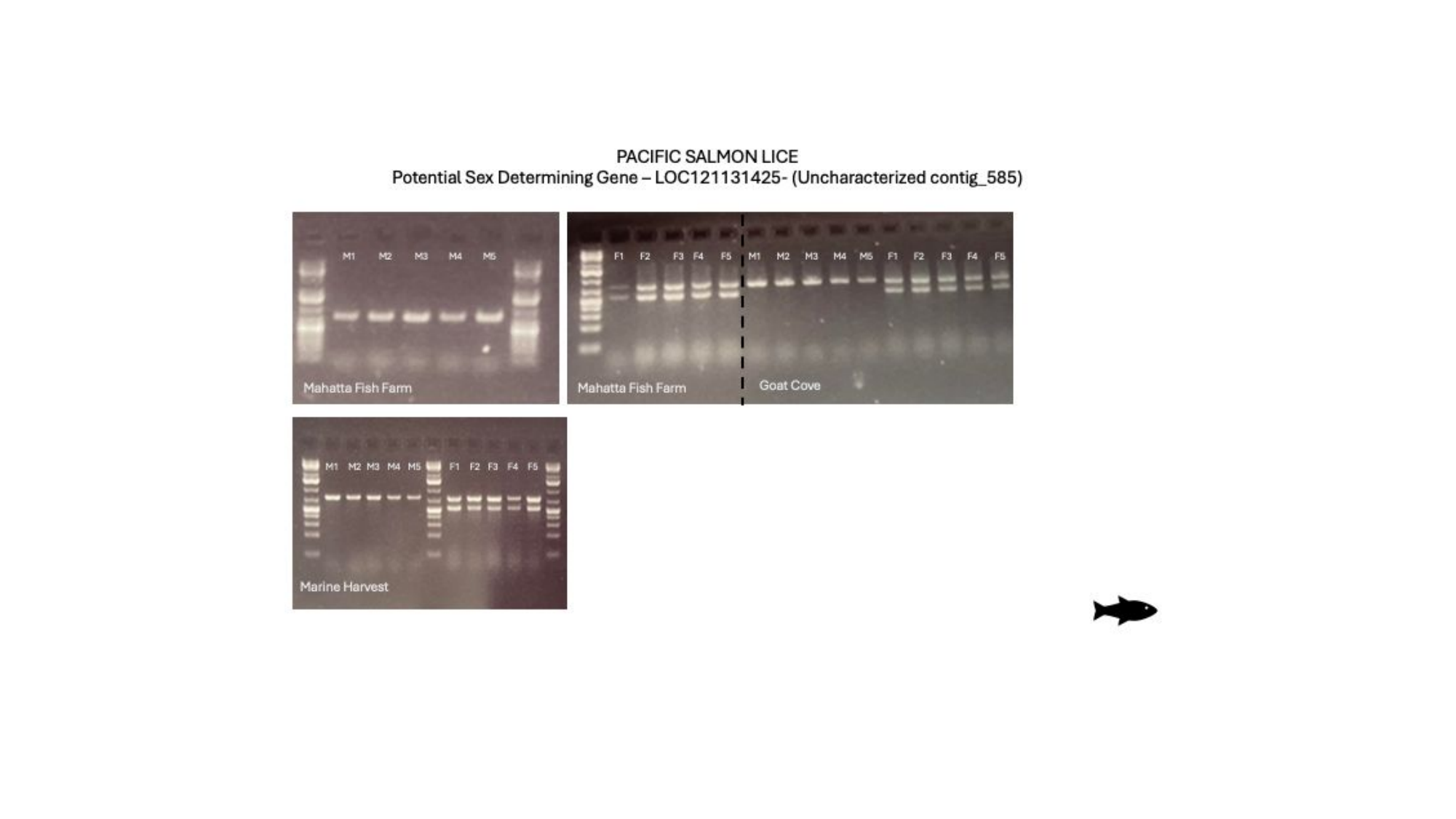

## Slide 4
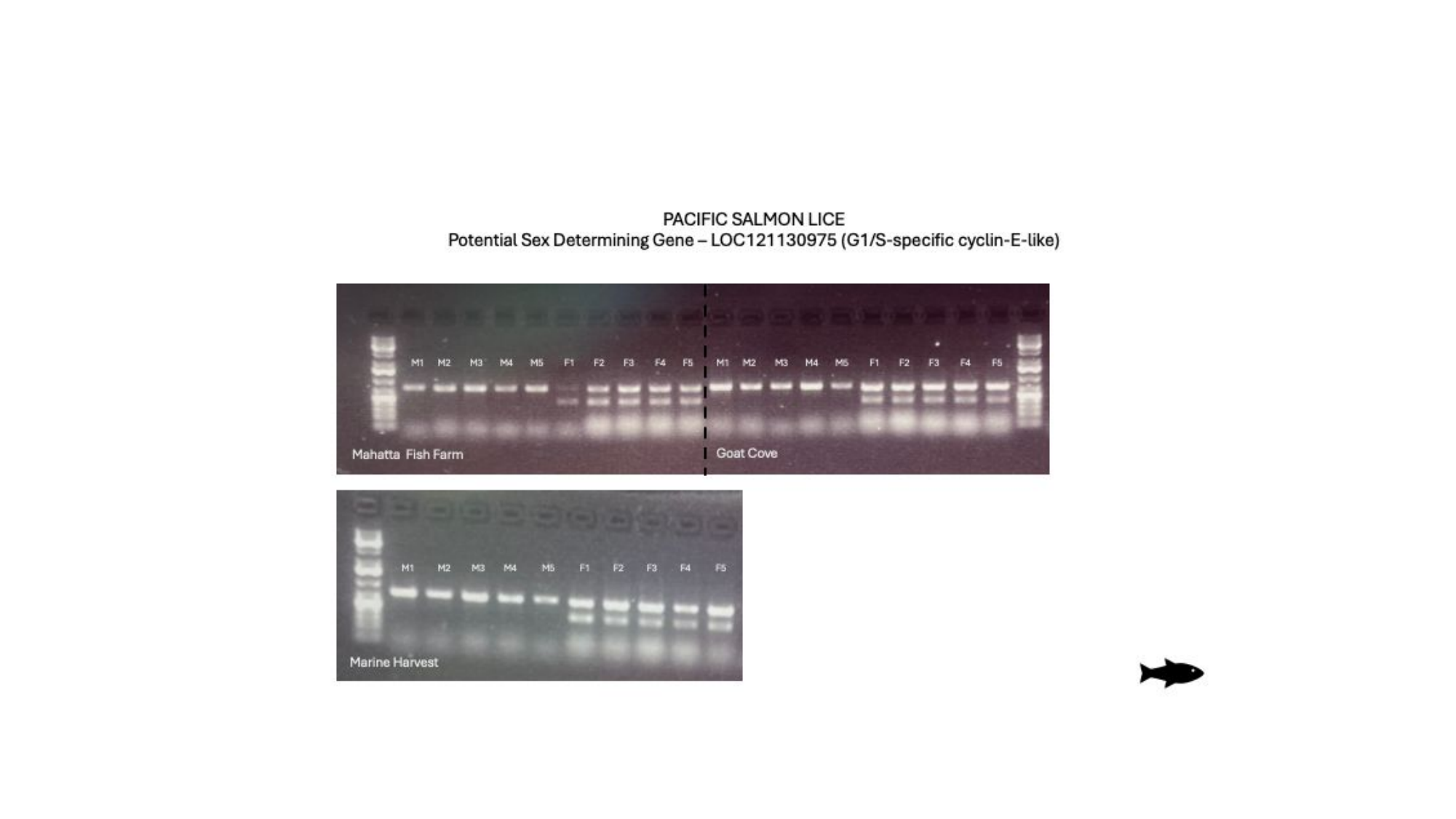

## Slide 5
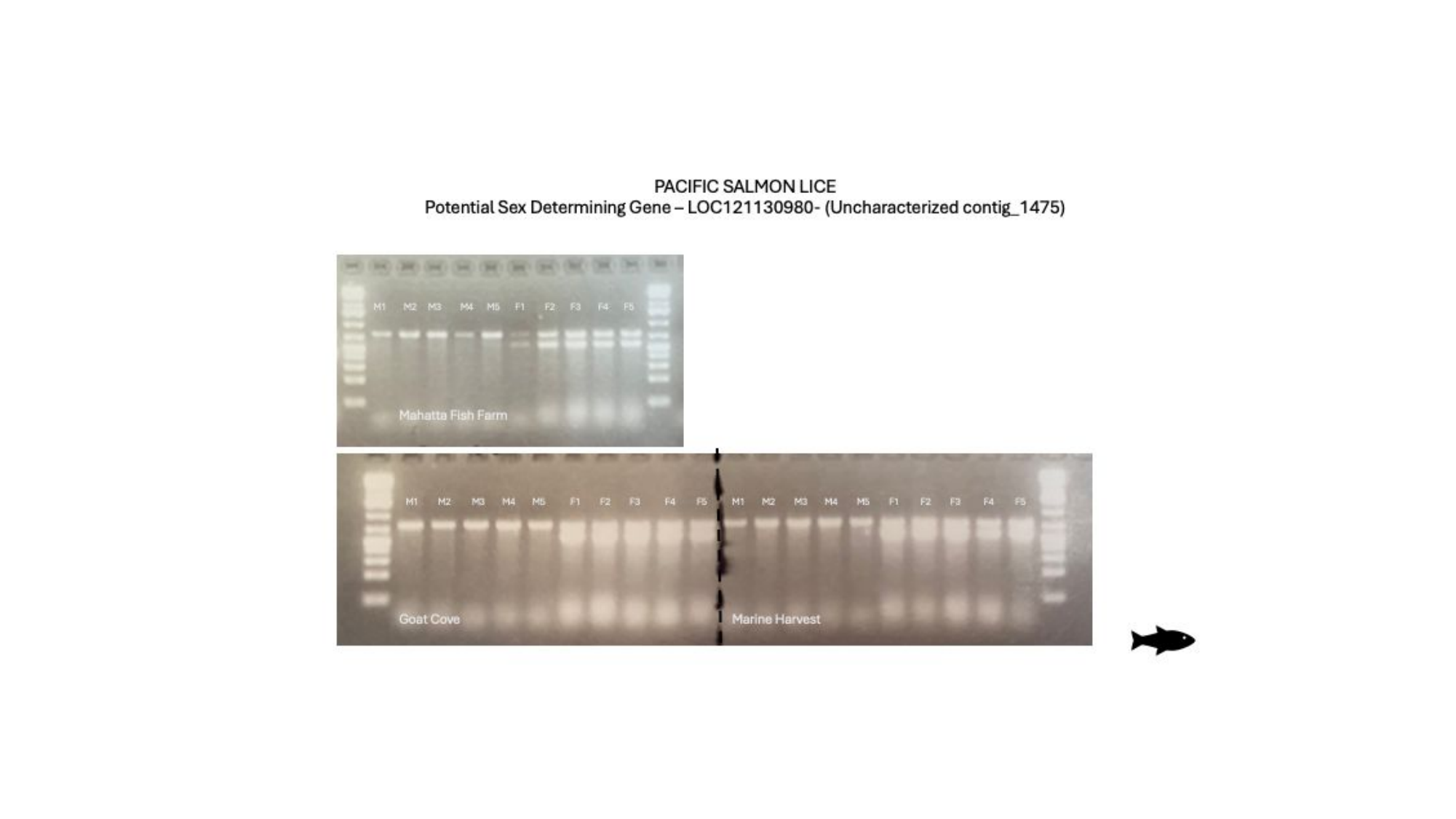

## Slide 6
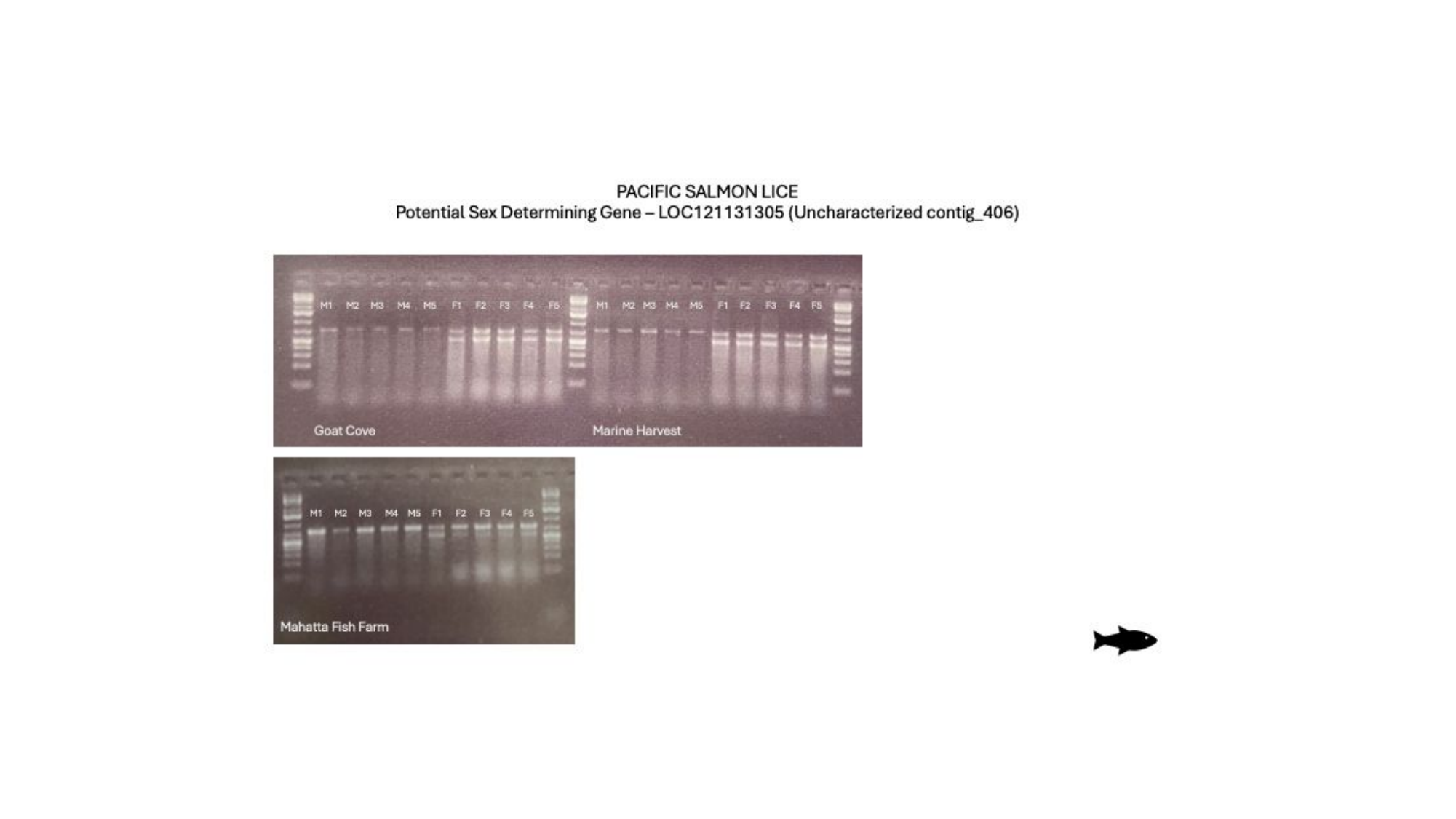

Supplement: jkae087_Supplementary_Data [file jkae087_supplementary_data.zip › File_S2_G3-2024-404899.pptx]
